# Gamma secretase dependent release of the CD44 cytoplasmic tail upregulates IFI16 in *cd44^-/-^* tumor cells, MEFs and macrophages

**DOI:** 10.1371/journal.pone.0207358

**Published:** 2018-12-12

**Authors:** Kristin Schultz, Christina Grieger (Lindner), Yong Li, Pavel Urbánek, Anne Ruschel, Kerstin Minnich, Dunja Bruder, Marcus Gereke, Antonio Sechi, Peter Herrlich

**Affiliations:** 1 Helmholtz Centre for Infection Research, Immune Regulation Group, Braunschweig, Germany; 2 Otto-von-Guericke-University Magdeburg, Institute of Medical Microbiology, Infection Prevention and Control, Magdeburg, Germany; 3 Leibniz Institute on Aging, Fritz Lipmann Institute (FLI), Jena, Germany; 4 Institute of Biomedical Engineering, Dept. of Cell Biology, Aachen, Germany; University of Colorado Denver School of Medicine, UNITED STATES

## Abstract

The adhesion molecule and co-receptor of receptor tyrosine kinases, CD44, is expressed in all cells of the immune system, but also in numerous non-immune cells. CD44 plays roles in the cellular response to different pathogens. The molecular actions of CD44 during these processes are by and large still unknown. The CD44 molecule undergoes a sequential proteolytic cleavage which leads to the release of a soluble intracellular domain (CD44-ICD). Previous reports had shown that the CD44-ICD is taken up into the nucleus where it enhances transcription of specific target genes. By RNA profiling we identified a CD44-dependent transcriptional increase of interferon-responsive genes, among them IFI16. IFI16 is important in the innate immune response. It senses and binds pathogenic DNA and, together with cGAS, activates the cGAS-cGAMP-STING pathway and induces the expression of genes relevant for the response, e.g. IFN-β. Our results show that the enhancement of IFI16 expression depended on CD44 cleavage. A CD44-negative tumor cell line, embryonic fibroblasts and bone marrow-derived macrophages from *cd44*^*-/-*^ mice were reduced in their response to IFN-γ, to viral DNA fragments and to *Listeria monocytogenes* infection. We could rescue the deficiency of CD44 negative RPM-MC cells and *cd44*^*-/-*^ MEFs by expressing only the soluble CD44-ICD in the absence of any other CD44 domain. Expression of the CD44-ICD carrying a mutation that prevented the uptake into the nucleus, could not rescue the absence of CD44. This molecular aspect of regulation by CD44 may explain part of the immune phenotypes of mice with *cd44* gene disruption.

## Introduction

**Fig 1:**
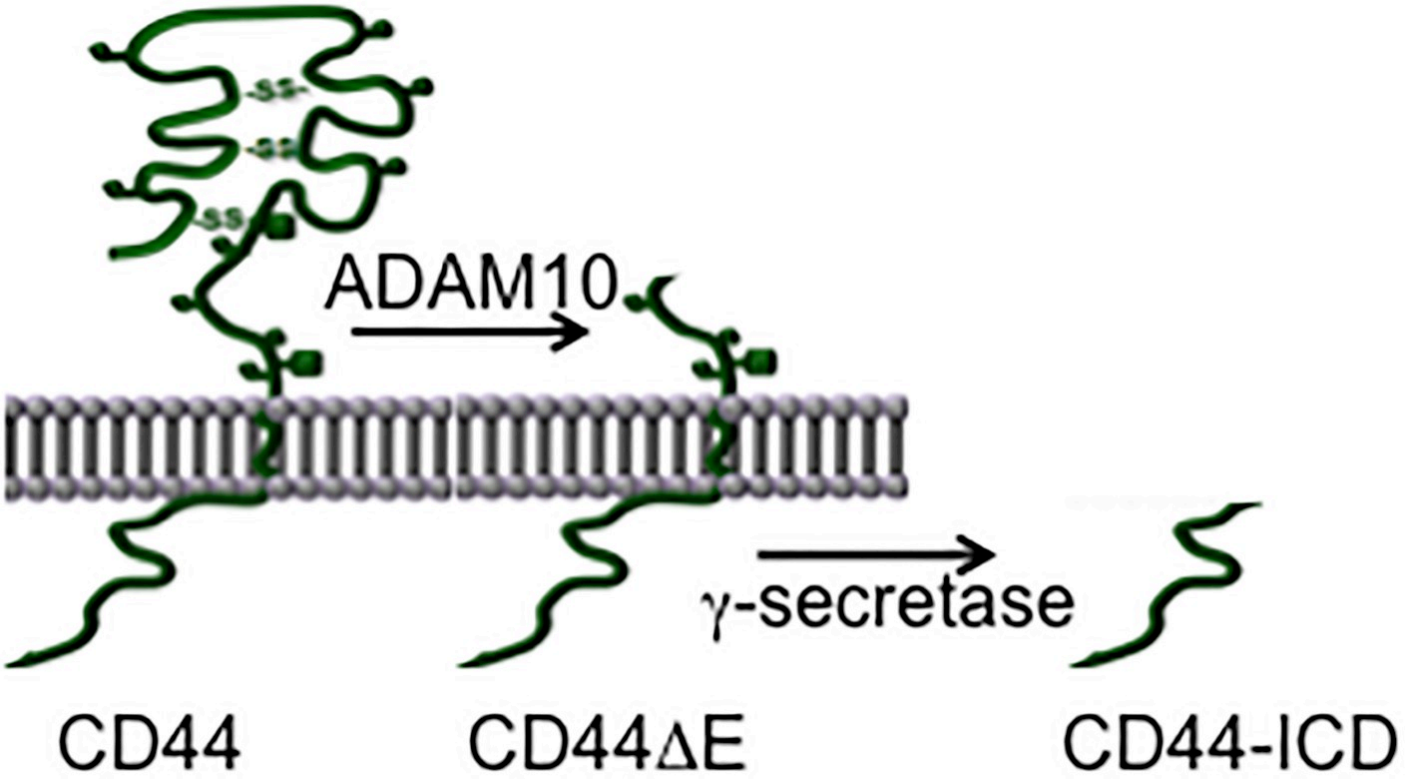
Schematic representation of CD44 cleavage induced by phorbol ester (TPA). The first cleavage step is mediated by membrane-associated matrix metalloproteases (predominantly by ADAM10) and leads to the release of the ectodomain into the extracellular space. The remaining CD44ΔE fragment is cleaved by γ-secretase which leads to the release of the intracellular domain (CD44-ICD) inside of the cell.

The transmembrane glycoprotein isoforms designated CD44 (cluster of differentiation 44) are encoded by a single gene whose transcript is subject to alternative splicing. Most abundantly expressed is the smallest splice form (CD44s). Alternative splicing alters predominantly the ectodomain of CD44 in that variant exons add sequences to its membrane-proximal stem structure. Like many other membrane proteins CD44 is subjected to regulated ectodomain shedding by a metalloprotease (in case of CD44 by ADAM10; [1–4]) and subsequent release of the intracellular domain (CD44-ICD) by γ-secretase [4–6] (see Fig 1). The CD44-ICD is taken up into the nucleus where it influences transcription, e.g. driven by AP-1, CREB or NF-κB [1,5,7].

Prominent sites of CD44 expression are the cells of the immune system. Absence of the gene exerts only a mild immune phenotype in non-challenged mice [8,9], but causes diverse effects on immune responses to infection [10–19]. The diversity of the organismic reactions to infection is likely due to molecular CD44 actions specific for cell type (and perhaps different CD44 isoforms), to different immune cells and other cell types involved in the responses, and to the interactions between different and multiple cell types (all possibly affected by deletion of CD44) during an immune response.

The complexity of CD44 and its isoforms in the complete organism makes a mechanistic study difficult. To reduce the complexity, we address here whether the cleavage of CD44 is relevant for its role in activating immune response relevant genes. We compared transcription induced by different physiologic and pathogenic agents in CD44-negative tumor cells, murine embryonic fibroblasts (*cd44*^*+/+*^ and *cd44*^*-/-*^ MEFs) and isolated bone marrow derived macrophages from *cd44*^*+/+*^ and *cd44*^*-/-*^ mice (in the absence of other cell types). We focused on the nucleic acid sensor IFN-γ inducible protein 16 (IFI16; Gene ID: 3428; IFI204 in the mouse, Gene ID: 15951) and its downstream target IFN-β. CD44-negative cells were reduced in their transcriptional response to interferon gamma (IFN-γ), viral DNA or infection with *L*. *monocytogenes*. We then asked with which domain of CD44 the absence of the gene could be compensated. The result was: The IFI16 dependent functions of CD44-negative cells were restored by expressing only the soluble CD44-ICD.

## Materials and methods

### Reagents

γ-secretase inhibitor N-[N-(3,5-Difluorophenacetyl)-L-alanyl]-S-phenylglycine t-butyl ester (DAPT; Enzo Life Sciences, Lörrach); phorbol-12-myristate-13-acetate (TPA) and Actinomycin D (Sigma–Aldrich, St. Louis); human IFN-γ (Thermo Scientific, Rockford); Lipofectamine 2000 (Invitrogen, Darmstadt); batimastat (Sigma–Aldrich, St. Louis).

### Cell culture

Primary embryonic fibroblasts were obtained from E12.5 embryos and cultivated in Dulbecco’s modified Eagle’s medium (DMEM, Gibco 11995–065) containing gentamycin (Gibco 15710–064), glutamine (Gibco 25030–149), MEM non-essential amino acids (Gibco 11140–050), 2-mercaptoethanol (Gibco 21985–023) and 10% fetal bovine serum (FBS) for spontaneous immortalization (MEFs). The CD44-negative human melanoma cell line RPM-MC [2–4] and MEFs immortalized from wild type mice or CD44 complete knockouts were grown at low cell density (3x10^5^ cells/ 6 cm dish for RPM-MCs and 1.5x10^5^ cells/ 6 cm dish for MEFs) in DMEM (PAA Laboratories GmbH, Pasching, Austria) supplemented with 10% fetal bovine serum (PAA Laboratories GmbH, Pasching, Austria). Knockout of CD44 was achieved by deleting exon 3 of CD44 in ES cells, which causes a frameshift and thereby a premature stop codon, and injection of the targeted ES cell into blastocysts to generate *cd44*^*-/-*^ mice.

Primary bone marrow-derived macrophages (BMDM) were isolated from 8 to 15 weeks old mice using standard protocols [20]. Briefly, isolated bone marrow cells were seeded into 6-well plates and differentiated along the macrophage lineage for six days prior to experiments in DMEM (high glucose), with 10% heat inactivated FBS, 1 mM sodium pyruvate, 2 mM L-glutamine, Penicillin-Streptomycin (Sigma P0781) and 17.5% L929 cell conditioned media (LCCM) as a source for macrophage colony-stimulating factor (M-CSF), or in RPMI 1640 with 20% L929 cell conditioned media (LCCM). On day 7 the medium was changed to 0.5 to 2% FBS, respectively, with all other incredients as above. Experimental use of the macrophages on day 8. For this, the macrophages were stimulated with or without 12,5 or 5 ng/ml IFN-**γ** and for later experiments IFN-**γ** with or without DAPT, or DAPT and batimastat.

### Plasmids and transfections

All experiments within this study which required safety level 2 were registered with and permitted by the Thüringer Landesverwaltungsamt (TLVwA) under the reference numbers: 22-016-012-14/wA and 22-016-012-15/wA.

Construction of the CD44KR mutant: CD44s in the pcDNA3 vector (Invitrogen, DeShelp) was mutated by PCR in the following positions: alanine substitutions for arginines at positions 293 and 294 and for lysines at positions 298, 299 and 300. A C-terminal Myc epitope tag was added. The primers used have been described in [21]. The PCR product was subcloned into the HindIII / XhoI sites of pcDNA3.1/Hygro(+) (Invitrogen). The properties of the CD44KR mutant have been described in ref. 4; CD44ΔE (gift of Christoph Kaether, FLI) has been described in ref. 5; all other CD44 plasmids and the lentiviral constructs have been described in [21], with one exception: human CD44-ICD was subcloned into the lentiviral vector pCDH-CuO-MCS-EF1-copGFP using primers 3 and 4 (Table 1). Cells were transfected with Lipofectamine 2000.

**Table 1:** Oligonucleotide sequences.

No.	Name	Sequence (5´-3´)
		**Oligos for cloning**
**1**	hCD44 fwd	gcggctagccagggatcctccagctcctttc
**2**	hCD44 rev	gcgaagcttccctgtaatggttatgtttccaacg
**3**	hCD44ICDCumatefwd	gcgtctagagccgccaccatggcagtcaacagtcgaagaaggtgt
**4**	hCD44Cumate rev	gcggctagcccctgtaatggttatgtttccaacg
		**VACV70mer Oligos**
**5**	VACV70mer fwd	ccatcagaaagaggtttaatatttttgtgagaccatcgaagagagaaagagataaaacttttttacgact
**6**	VACV70mer rev	agtcgtaaaaaagttttatctctttctctcttcgatggtctcacaaaaatattaaacctctttctgatgg
		**Oligos for qRT-PCR**
**7**	β-Actin fwd	agagggaaatcgtgcgtgac
**8**	β-Actin rev	caatagtgatgacctggccgt
**9**	hIFI16-RTF4	acttcatgaggatgcagatactg
**10**	hIFI16-RTR3	gaggtcactctgggcactgtc
**11**	mIFI16-RTF1	gggggacatttgtgagtggagagta
**12**	mIFI16-RTR1	tactgcctggttcacacctgacat
**13**	mIFN-β fwd	atggtggtccgagcagagat
**14**	mIFN-β rev	ccaccactcattctgaggca
**15**	miNOS fwd	ggcagcctgtgagacctttg
**16**	miNOS rev	gcattggaagtgaagcgtttc
**17**	CXCL10 fwd	gctgccgtcattttctgc
**18**	CXCL10 rev	tctcactggcccgtcatc
**19**	CXCL11 fwd	gctgctgagatgaacaggaa
**20**	CXCL11 rev	ccctgtttgaacataaggaagc
**21**	Arg1 frd	ctttctcaaaaggacagcctcg
**22**	Arg1 rev	cacagaccgtgcgttcttca
**23**	Ptgs2 frd	catccccttcctgcgaagtt
**24**	Ptgs2 rev	ctccttatttcccttcacaccca
**25**	TNFα frd	tagcccacgtcgtagcaaac
**26**	TNFα rev	gcagccttgtcccttgaaga
**27**	β-Act frd (2)	cttctttgcagctccttcgt
**28**	β-Act rev (2)	Tccttctgacccattcccac

For lentiviral transduction, HEK293T cells at 90% confluence were transfected with the plasmid of interest together with the packaging plasmid (pCMVΔR8.91) and the VSV-G envelope plasmid (pCMV-VSVG or pMDG-VSVG) in a ratio of 2:1:1. Cells were incubated for 5 to 8 hrs at 37°C and then changed to 32°C for O/N incubation. The next morning medium was refreshed and cells were again incubated O/N. Then, the supernatant was harvested with a 2-ml syringe (Braun, Melsungen, Germany) and filtered onto target cells through a 0.45 μM sterile filter (Sarstedt, Nümbrecht, Germany). Desired target cells for transduction were seeded into 6-well plates and transduced after reaching a confluence of 25 to 50%. 24 hrs after transduction, cells were washed 2 to 3 times with fresh medium and cultured for two days prior to experiments.

### Animal maintenance

Mice were maintained in IVC cages with individual ventilation. A veterinarian routinely supervised the condition of animal handling. Hygiene was controlled according to the FELASA guidelines. All material entering the animal facility was autoclaved or chemically disinfected. Conditions in the mouse rooms: 21+/-2°C, 10–15 x air exchange per day, 12h/12h day/night cycles with 20 min dimming period.

### RNA Isolation and quantitative real-time PCR

RNA was isolated using the RNeasy Mini Kit (Qiagen, Hilden, Germany). Subsequently 1 μg of total RNA was reverse-transcribed with oligo(dT) primers and 200 U of Superscript (Invitrogen GmbH, Darmstadt, Germany). RNA quality was controlled via gel electrophoresis and β-actin PCR. cDNA was analysed by qPCR (Sybr Green, Invitrogen) using primers specified in Table 1. The qPCR results were standardized first to actin, and subsequently to non-treated cellular controls.

### Treatment of cells

To induce IFI16 mRNA, cells were serum-starved in medium containing 0.5% FBS (unless otherwise stated) for 24 hrs prior to stimulation. To mimic a viral infection, cells were transfected with a 70 bp long repetitive double-stranded (ds) sequence from the *vaccinia virus* genome (VACV70mer, [22]). The ds VACV70mer was generated by annealing two single stranded oligos (Table 1, No. 5 and 6). 1 μg/ml VACV70mer were transfected into cells using Lipofectamine 2000 for 6 hrs (as described in [21]). For transfection reactions the medium volume per 6 cm dish was reduced from 5 to 2.5 ml.

BMDMs on day 8 as described above were stimulated with 5 ng/ml murine IFNγ (PeproTech) with or without 5μM DAPT +/- 5μM batimastat, or non-stimulated. RNA was isolated after 6 hours, and its concentration measured by Nanodrop. Transcription into cDNA as described above.

For *Listeria monocytogenes* infection experiments, BMDMs were isolated and differentiated as describe above. The evening before infection, 50 ml BHI broth was inoculated with few *L*. *monocytogenes* colonies. The strain was grown O/N at 37°C on an orbital shaker (neoLab, Heidelberg, Germany) in Nalgene Sterile Single Use Erlenmeyer Flasks, Vented Closure, 500 ml (Thermo Scientific, Rockford, IL, USA). By the time the bacteria reached an OD 600 of 2, they were used for infections. In a first step, the bacteria were washed with PBS (3000 rpm, 5 min) and resuspended in 50 ml. The bacteria were diluted 1:10.000 or 1:5.000 in infection medium (3 ml infection medium per 6 cm dish was used) and distributed over the dishes with BMDMs. Dishes with cells were centrifuged (2000 rpm, 5 min) in order to synchronise the infection process, and incubated for 1 h at 37°C. Subsequently, BMDMs were washed with post-infection medium (DMEM with P/S and 15 μg/ml gentamycin). Addition of gentamycin prevented extracellular bacterial growth. Afterwards, BMDMs were returned to the incubator for time periods as indicated.

### Statistical analysis

Statistical significance was determined using Student’s t-test. The data are expressed as the mean±SEM. A *p*-value of less than 0.05 was considered statistically significant. All experiments were done in triplicates and repeated at least three times. * = < 0.05, ** = < 0.01, *** = < 0.001.

## Results

### CD44 enhances transcription of the interferon-inducible protein 16 (IFI16)

**Fig 2:**
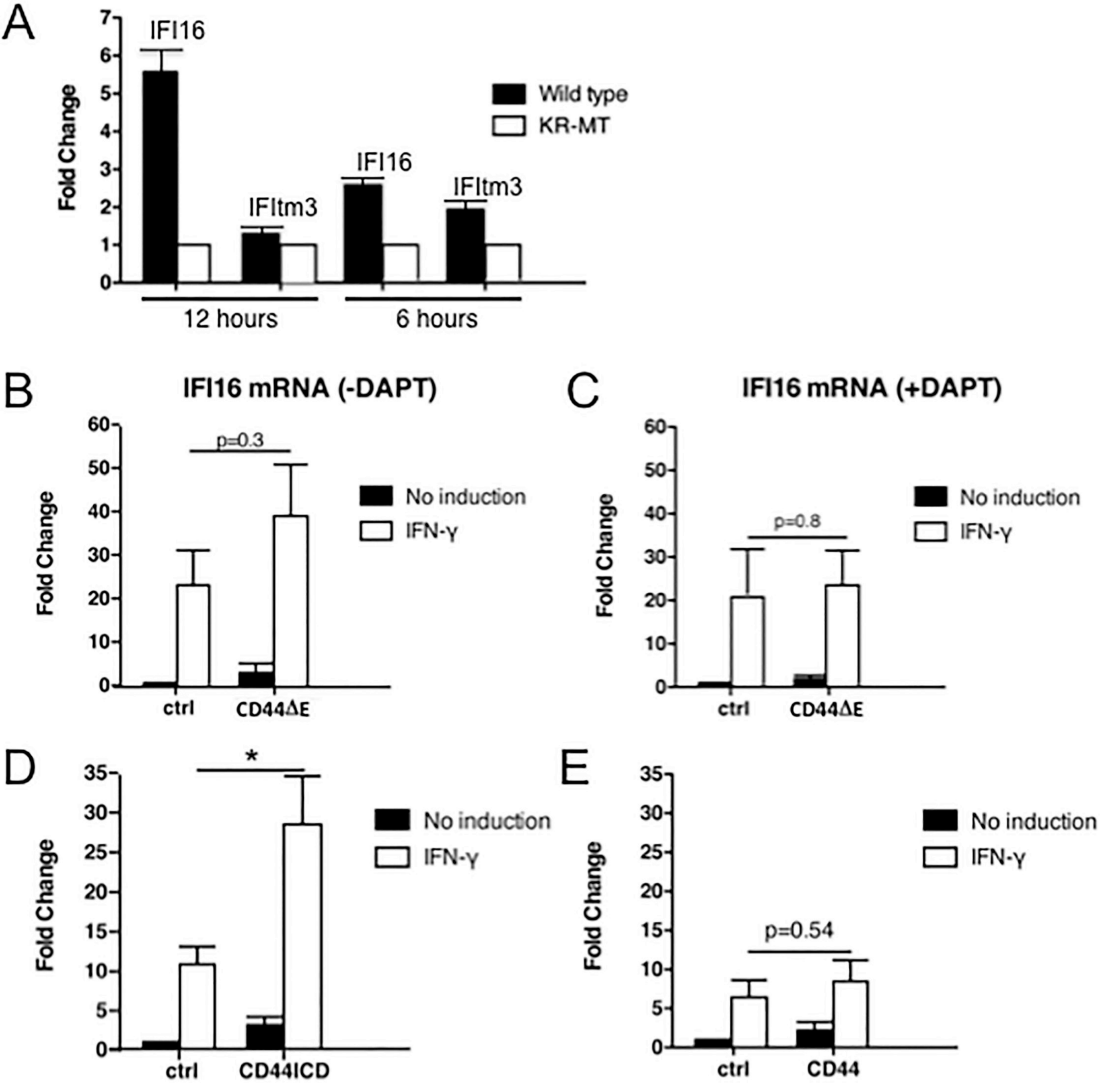
CD44 cleavage is required for IFN-γ-induced expression of IFI16. **(A**) RPM-MC cells were transfected either with full-length CD44WT or CD44-KR-MT, serum starved O/N and subsequently treated with 100ng/ml TPA for 30 min. After additional 12 or 6 hrs of cultivation in serum-free medium, total RNA was isolated and used for RT-qPCR. Similar results were obtained in 3 separate experiments (IFI16: interferon-inducible protein 16: IFItm3: interferon-inducible transmembrane protein 3). (**B-E**) RPM-MC cells were seeded in 6 cm dishes at a cell density of 3x10^5^ cells/dish. 24 h after seeding, cells were transiently transfected with either a control vector or with vectors expressing either CD44ΔE (mimicking the product of ADAM cleavage, which serves as substrate for γ-secretase), CD44-ICD or CD44 full length. For cell recovery, medium was changed after 14 h. Subsequently, cells were stimulated with 100 ng/ml IFN-γ for 6 h. RNA was isolated and transcribed into cDNA. cDNA was analysed by RT-qPCR using primers for human IFI16 (primers 9 and 10, Table 1) and actin for normalization (primers 7 and 8, Table 1). In Fig 2C γ-Secretase activity was blocked by 5 μM DAPT O/N prior to IFN-γ treatment. All experiments were done in triplicates. For statistical analysis see Materials and methods.

CD44 is a transmembrane protein which can induce transcription through signaling pathways (e.g. [23,24,25]). To compare the gene expression profiles of cells in the absence or presence of CD44, we transfected into CD44-negative RPM-MC cells CD44s (full-length, wild type, the smallest and most widely expressed isoform) or CD44-KR-MT (described in [4]), a mutant in the binding site for ERM proteins thought to be required for signaling to the nucleus through Ras activation [23–28], as well as for regulation of ectodomain cleavage [2,3]. The mutation is located in the cytoplasmic tail and eliminates several of the charged amino acids. We compared the RNA profiles by microarray. In the CD44s-transfected cells the expression of several genes was higher than in the cells supplied with CD44-KR-MT (not shown). Among these were genes of relevance for the innate immune system, e.g. interferon-inducible genes. We confirmed their inducibility by RT-qPCR. The presence of CD44s enhanced the phorbol ester (TPA) induced transcription of interferon-inducible protein 16 and of IFNγ-inducible transmembrane protein 3 (IFItm3) compared to cells expressing CD44-KR-MT (Fig 2A). The induction kinetics of the two transcripts appeared to be different, with IFItm3 RNA detectable earlier than IFI16 RNA. Because of the stronger inducibility and the significance for viral and bacterial cellular defense we followed IFI16 expression in subsequent experiments.

### Enhanced IFI16 transcription requires CD44 cleavage

**Fig 3:**
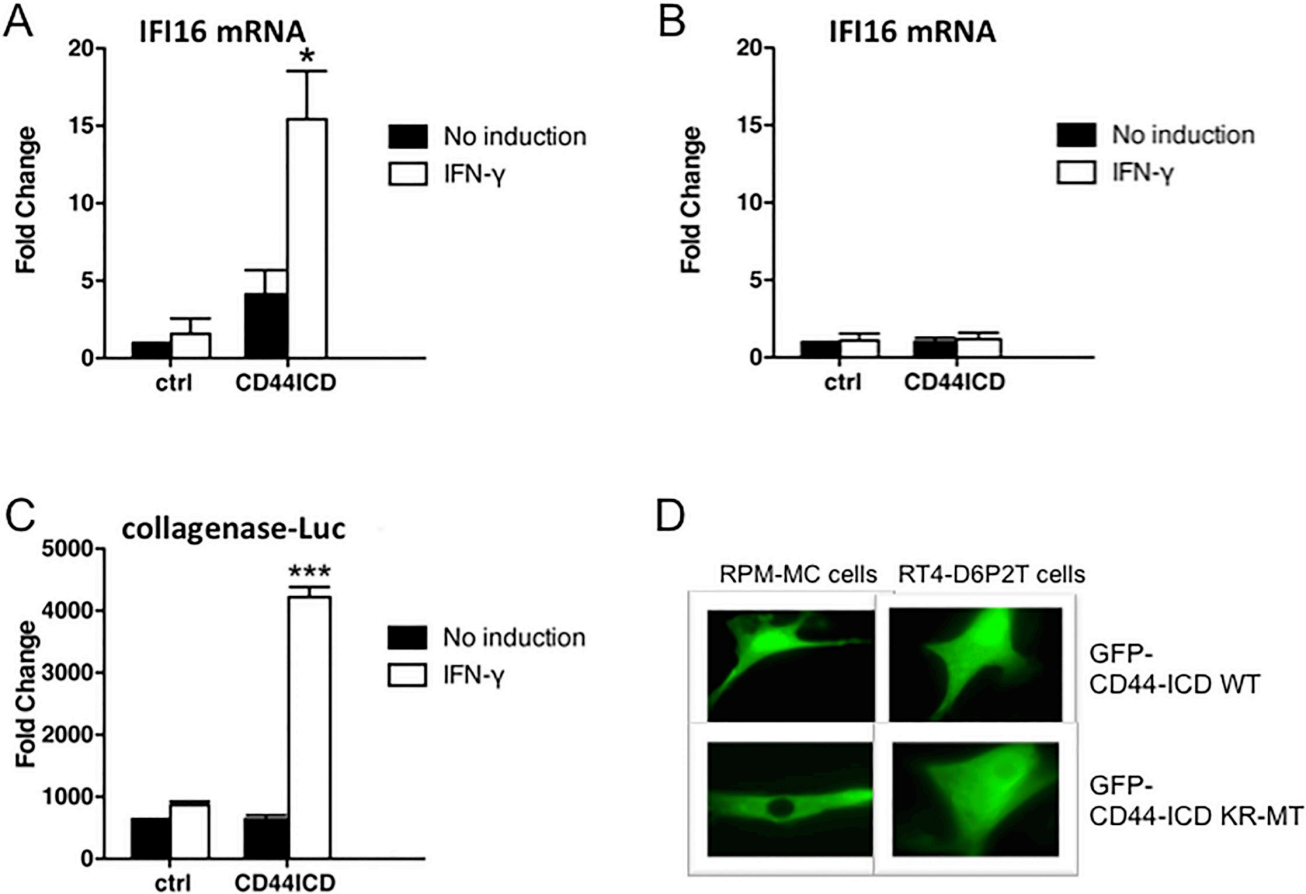
IFI16 up-regulation is implemented at the level of transcription. **(A**) RPM-MC cells were prepared as in Fig 2 and transfected with a control vector or a vector expressing the CD44-ICD as described above. (**B**) Immediately before treating cells with IFN-γ (100 ng/ml, 6 h), actinomycin D (5 μg/ml) was added to the cells. RNA was analysed as described in Fig 2. (**C**) RPM-MC cells were seeded in 24-well plates at a density of 4x10^4^ cells/well. 24 h later cells were transiently transfected with either a control vector or CD44-ICD together with a construct of collagenase 1 promoter fused to the firefly-luciferase gene. Transfection medium was exchanged to fresh medium after 14 h. Overnight treatment with IFN-γ (100 ng/ml) was followed by analysis. Luminescence was measured after addition of Firefly-luciferase substrate. (**D**) Cells were transiently transfected with GFP-tagged wild type or KR-MT intracellular domain of CD44 (CD44-ICD). After 24 h, cells were fixed with 4% PFA and examined by microscopy (Axiovert 135). RPM-MC cells: 46 cells of total 50 cells showed nuclear localisation of wild type CD44-ICD; 32 cells of total 50 cells showed cytoplasmic localization of mutant CD44-ICD. RT4-D6P2T cells: 45 cells of total 50 cells showed nuclear localisation of wild type CD44-ICD; 30 cells of total 50 cells showed cytoplasmic localization of mutant CD44-ICD. Experiments were done in triplicates. For statistics see Materials and methods.

There are a number of options how CD44s could enhance transcription: i) the ectodomain could mediate a co-receptor function for cytokines or growth factors [23]; ii) even in the absence of the ectodomain, the membrane-bound truncated version carrying the cytoplasmic domain could signal to levels of expression [21]; iii) the released ICD upon cleavage could enhance transcription [5–8]. By co-immunoprecipitation through pan-CD44-antibodies we identified two importins attached to CD44 (unpublished), suggesting uptake of either the complete CD44 molecule or parts of it into the nucleus. We have shown previously that the CD44-KR-MT mutant [4] was defective in signal transduction [23] as well as in induced cleavage by ADAM10 [4]. If only release of the ectodomain by ADAM10 were decisive, a CD44 truncation mimicking the result of ADAM cleavage (no ectodomain, CD44ΔE, see Fig 1) should not rescue the induced expression. CD44ΔE however enhanced IFN-γ-induced transcription of IFI16 (Fig 2B). Proteolysis by ADAM is a prerequisite for subsequent processing: CD44ΔE serves as substrate for the subsequent cleavage reaction by γ-secretase. Inhibition of γ-secretase by DAPT abolished the enhancement (Fig 2C). Apparently complete cleavage was required. To directly prove that it is the released ICD and not the residual transmembrane domain that acted on IFI16 expression, we bypassed the cleavage reaction by expressing exclusively the ICD of CD44. The ICD indeed rescued the reduced expression of IFI16 due to the absence of the complete CD44 molecule (Fig 2D). Overexpression of CD44-ICD was more effective than expressing the CD44 full-length molecule (Fig 2E, compare with Fig 2D). It has been shown previously that the soluble CD44-ICD is transported to the nucleus where it exerts transcriptional stimulation through several transcription factors [5–8]. Indeed, the transfected GFP-tagged ICD wt molecule was detected in the nucleus (Fig 3D). Interestingly, the mutant protein, GFP-KR-MT-ICD, was not taken up into the nucleus (Fig 3D). It appears that the ICD requires active transport to the nucleus and the KR sequence not only serves to bind ERM proteins and to promote cleavage by ADAM10, but also serves as nuclear translocation motif. Addition of actinomycin D inhibited IFI16 induction (Fig 3B, compare with Fig 3A) indicating that the increase was due to transcription rather than mRNA stabilisation. The promoter region of IFI16 carries several AP-1 (Jun:Fos) target sequences which are required for IFI16 expression [29]. As expected, transfection of CD44-ICD stimulated the expression of another AP-1-dependent promoter, a collagenase-promoter fused to a firefly luciferase reporter (Fig 3C). Because Fos is limiting in most cells, we examined c-fos induction. Both TPA and IFN-γ induce c-fos expression (not shown), a limiting factor in the formation of active AP-1.

### CD44-ICD stimulates expression of IFN-β

**Fig 4:**
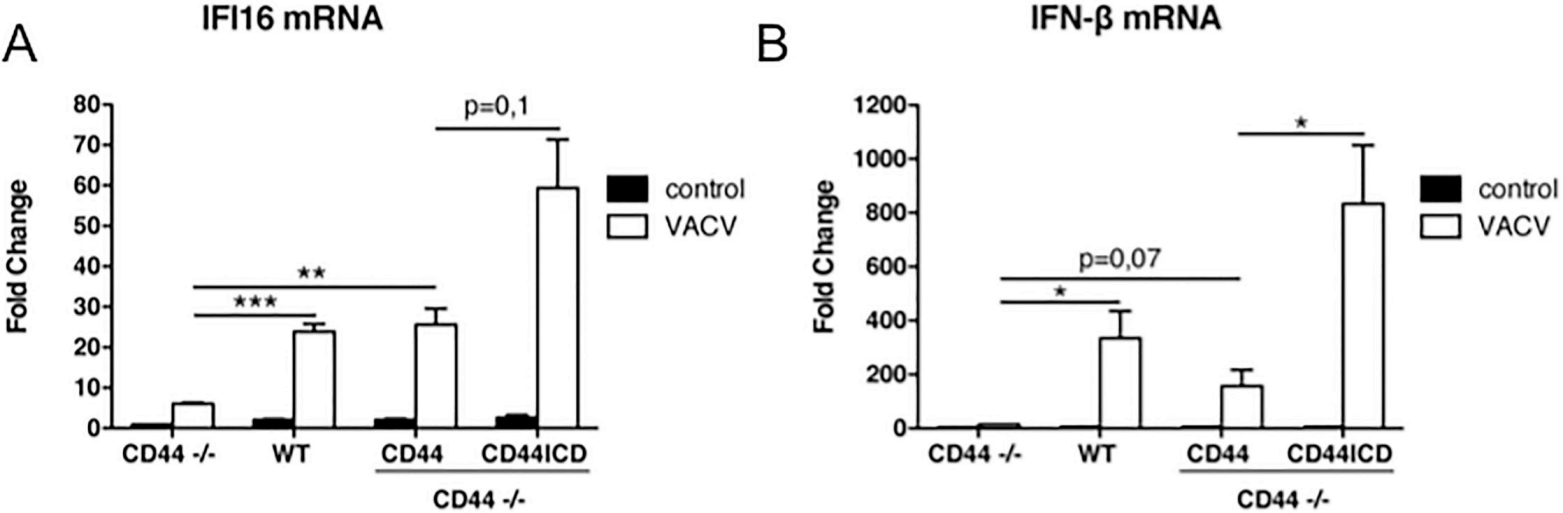
Rescue of viral DNA-induced IFI16 and IFN-β expression by CD44-ICD in cd44^-/-^ MEFs. MEFs from CD44 complete knockout mice were lentivirally transduced with either CD44 full length or CD44-ICD as described in Materials and Methods. Two days after transduction *cd44+/+* and *cd44-/-* MEFs were transfected with the VACV70mer oligo for 6 h as described in Materials and Methods. RNA was isolated and transcribed into cDNA. cDNA was analysed by RT-qPCR using primers for murine IFI16 (primers 11 and 12, Table 1) and IFN-β (primers 13 and 14, Table 1) and actin for normalization (primers 7 and 8, Table 1). Experiments were done in triplicates. For statistics see Materials and methods.

IFI16 protein is one of the nucleic acid sensing proteins which act through a number of intermediate steps involving STING and IRF3 [22,30,31,32]. Upon e.g. a viral infection IFI16 forms a transcriptional complex with other proteins. The complex activates IFN-β transcription as well as the expression of IFI16 itself [22]. To induce this nucleic acid sensing complex, we transfected a vaccinia DNA fragment (VACV70mer, [22]) into MEFs derived from *cd44*^*-/-*^ mice. The induction in *cd44*^*-/-*^ MEFs (without transduction of CD44) of both IFI16 and IFN-β by the VACV70mer was low, about 6-fold for IFI16 and 15-fold for IFN-β, (Fig 4, note the scale difference between Fig 4A and 4B). In WT MEFs (expressing the endogenous *cd44* gene) IFI16 expression was enhanced 24-fold (Fig 4A), IFN-β RNA 335-fold (Fig 4B). CD44s (full-length) transfection into *cd44*^*-/-*^ MEFs yielded a similar enhancement, although the induction of IFN-β RNA was only half of that in WT cells. We have no evidence whether the higher induction in WT cells was caused by the large variety of CD44 splice variants that could be expressed in the *cd44*^*+/+*^ cells. One could speculate that in the WT cells signaling pathways are activated in addition to the release of the ICD and that these address IFI16 and less or not IFN-β. The strongest induction of both, IFI16 and IFN-β, was achieved by transduction of only the CD44-ICD (Fig 4). We conclude that the CD44-ICD considerably enhances the activity of transcription factors at the IFI16 promoter upon induction by foreign DNA. Elevated IFI16 protein auto-stimulates its own synthesis and induces that of the target gene IFN-β.

### Impaired immune response *in vitro* of bone marrow derived macrophages from *cd44*^*-/-*^ mice

**Fig 5:**
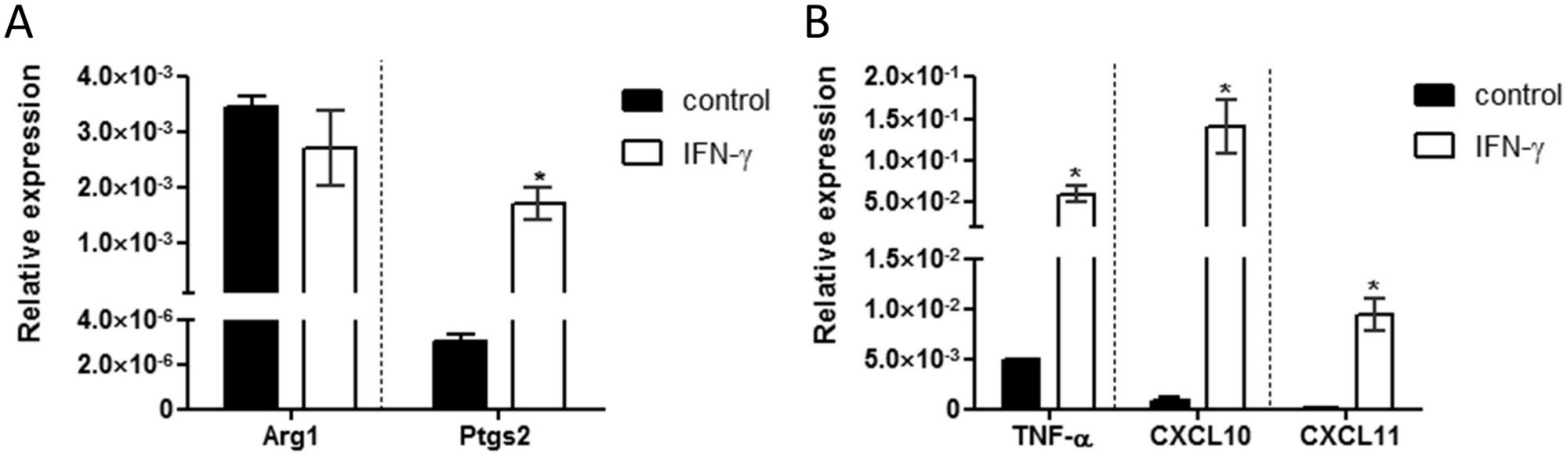
M1 macrophage phenotype after 6 hrs stimulation with IFN-γ. BMDMs were isolated from *cd44*^+/+^ control animals and differentiated. For differentiation 20% L929 conditioned media was used, with a reduction of FBS on day 7 to 2%. On day 8 macrophages were either not stimulated (control) or stimulated with 12,5 ng/ml IFN-γ for 6 hrs. After 6 hrs macrophages were harvested, RNA was isolated and analysed by RT-qPCR for (A) expression of the M1 macrophage gene Arg1, and of the M2 macrophage gene Ptgs2. (B) The RNAs were further analysed by RT-qPCR for the M1 specific cytokine TNF-α and M1 chemokines CXCL10 and CXCL11.

The regulation of interferon-responsive genes such as IFI16 indicates a role of the CD44 cytoplasmic tail in cells of the innate immune system. To confirm this notion, we explored the function of macrophages in the presence or absence of CD44. First, bone marrow precursor cells from WT mice were differentiated *in vitro* with or without IFN-γ stimulation for 6 hrs on day 8. RNA was isolated and analyzed by RT-qPCR for macrophage specific genes. Without IFN-γ a fraction of the differentiated macrophages expressed the typical M2 macrophage marker gene arginase 1 (Arg1, see [31]). Upon IFN-γ stimulation a slight reduction of Arg1 expression was observed (Fig 5A). Of note, the expression of Arg1 in M2 macrophages is considerably higher compared to control and M1 macrophages (not shown). IFN-γ stimulation caused a significantly increased expression of the typical M1 macrophage marker gene prostaglandin-endoperoxide synthase 2 (Ptgs2, see [31]), as well as high level expression of the typical M1 macrophage cytokines and chemokines tumor necrosis factor α (TNF-α), C-X-C motif chemokine ligands 10 and 11 (CXCL10 and CXCL11) (Fig 5A and 5B).

**Fig 6:**
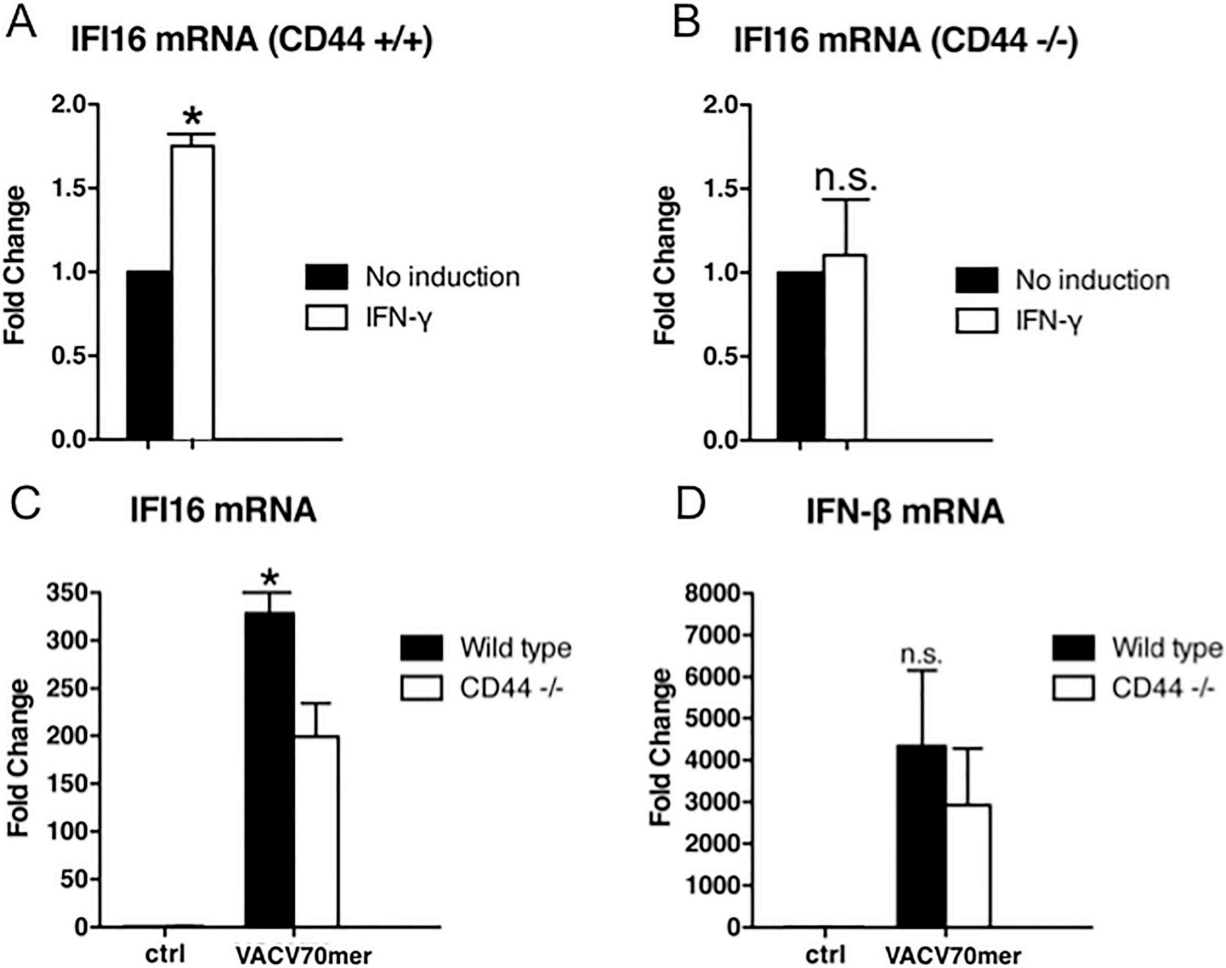
Impaired induction of IFI16 expression in cd44^-/-^ bone marrow-derived macrophages. Bone marrow-derived macrophages (BMDMs) from wild type and *cd44*^*-/-*^ mice were differentiated as described in Materials and Methods. Subsequently, the macrophages were serum-starved in macrophage medium w/o heat-inactivated FBS for 24 h and stimulated with 10 ng/ml IFN-γ for 6 h (A and B) or transfected with the VACV70mer oligo (C and D). RNA was determined as in Fig 4. Experiments were done in triplicates. For statistics see Materials and methods.

To now examine the dependence of IFI16 expression on CD44, bone marrow derived macrophages from WT and *cd44*^-/-^ mice were treated with IFN-γ and the transcription of IFI16 was monitored. A close to 2-fold induction was observed in wild type macrophages, but no response in macrophages from *cd44*^*-/-*^ mice (Fig 6A and 6B). In the absence of an extracellular stimulus (e.g. IFN-γ) transfection of the VACV70mer also strongly induced IFI16 as well as IFN-β in wild type macrophages while the induction was reduced in CD44 null macrophages (Fig 6C and 6D).

The innate immune response is part of the organismic defense against viral infections, but also against bacteria. Infection with *L*. *monocytogenes* is a prime example for the role of macrophages [33,34]. Infection with *L*. *monocytogenes* should trigger a response similar to that by viral DNA, also inducing IFI16, through DNA fragments or dinucleotides released [30,35].

**Fig 7:**
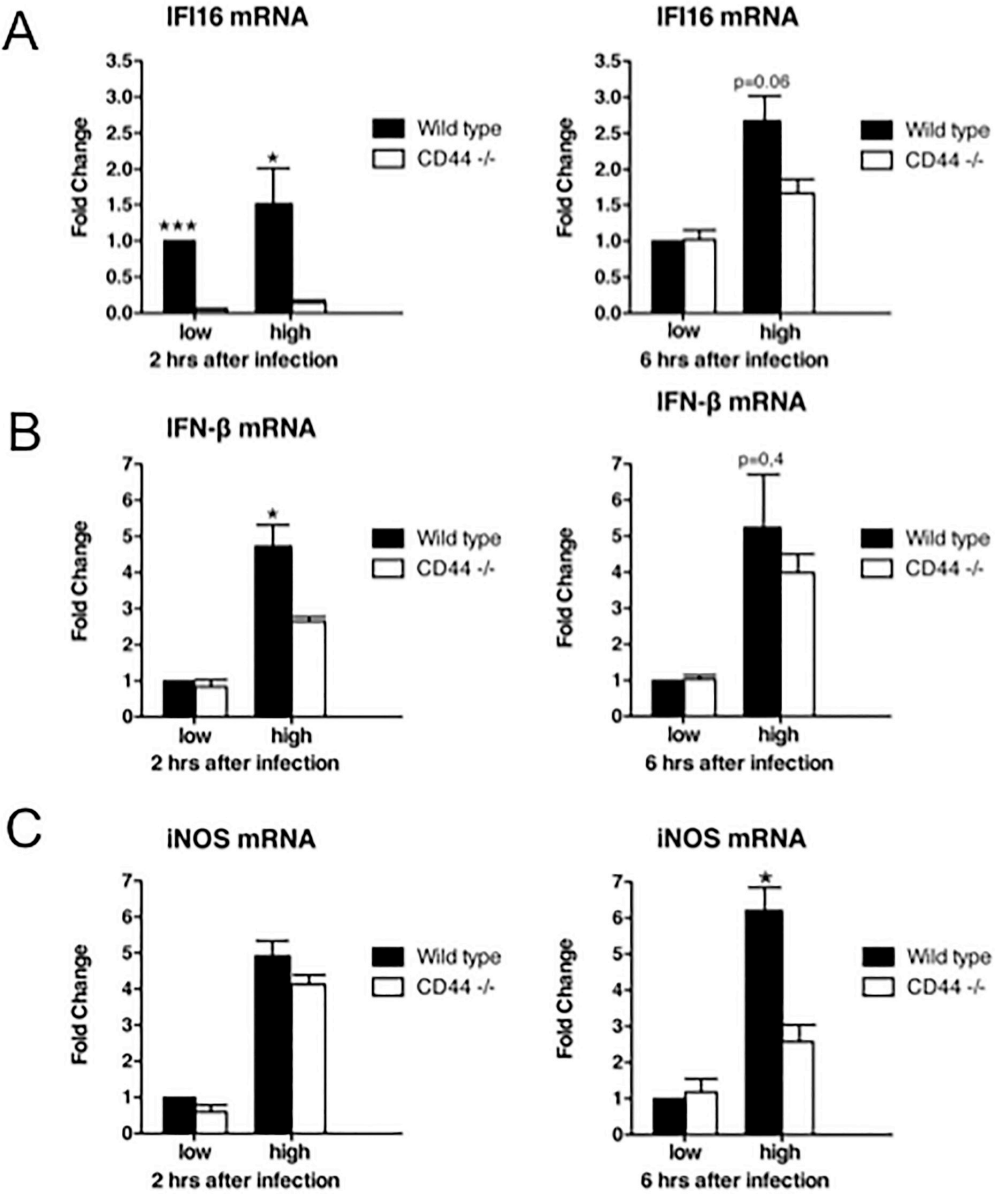
Impaired responses of cd44^-/-^ bone marrow-derived macrophages. BMDMs were isolated and differentiated from wild type control and knockout animals. *L*. *monocytogenes* were grown to an OD of ∼ 2.2. Bacteria were diluted 1:10.000 (low) or 1:5.000 (high) in infection media and introduced to macrophages as described in Materials and Methods. Cells were harvested 2 and 6 h after infection and analysed by RT-qPCR with **(A)** murine IFI16 oligos (primers 11 and 12, Table 1) or **(B)** IFN-β oligos (primers 13 and 14, Table 1) or **(C)** iNOS oligos (primers 15 and 16, Table 1). Experiments were done in triplicates. For statistics see Materials and methods.

Indeed, *Listeria* infection led to increased expression of both IFI16 and IFN-β in WT macrophages carrying CD44 (Fig 7A and 7B). *cd44*^*-/-*^ macrophages were defective or at least reduced in this response. The reduced response was particularly visible at early times after infection (compare 2 hrs and 6 hrs after infection in Fig 7A and 7B). The reaction to infection depended on the number of bacteria introduced into cells and was most pronounced after a high dose of bacteria.

Bacteria-infected cells are killed by nitric oxide (NO) and apoptosis triggered by reactive oxygen intermediates. To achieve apoptosis, IFN-β released by macrophages induces the expression of inducible nitric oxide synthetase (iNOS, [33]). Although *Listeria* can exploit the response rather than being killed [36,37], the induction of iNOS was still enhanced by the presence of CD44 (Fig 7C). The expression of iNOS reflected the induced expression of IFI16 and IFN-β; it was educed in *cd44*^*-/-*^ macrophages (Fig 7C). At 6 hrs after infection with *L*. *monocytogenes* the difference between CD44 positive and negative macrophages was most pronounced.

### Is CD44 cleavage required for IFI16 expression in differentiated macrophages?

**Fig 8:**
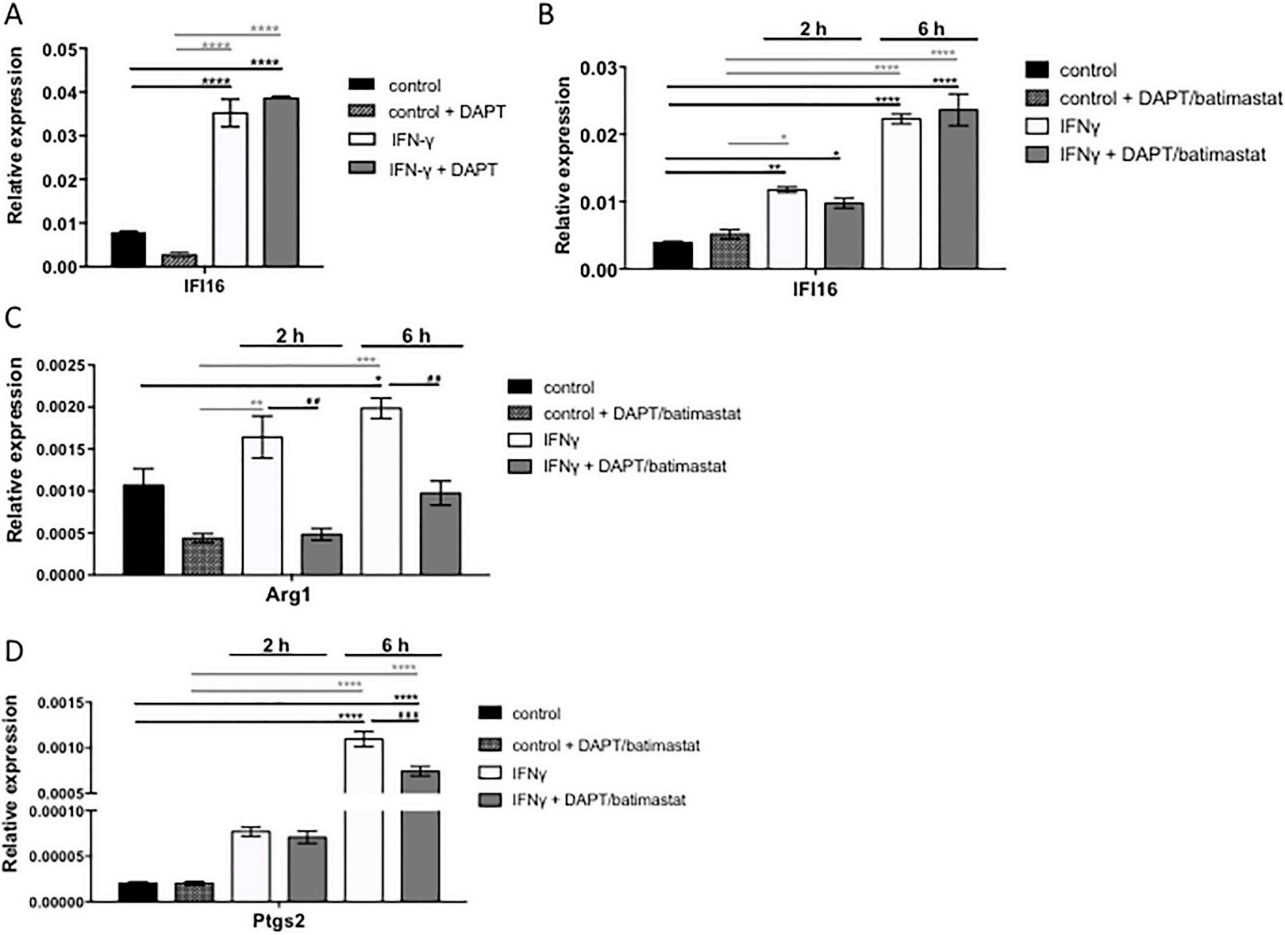
Influence of DAPT/batimastat on M1 macrophages. BMDMs were isolated and differentiated from *cd44*^+/+^ control animals. For differentiation 20% L929 conditioned media was used, with a reduction of FBS on day 7 to 2%. A) On day 8 macrophages were either not stimulated (control), stimulated with DAPT (control + DAPT); 12.5 ng/ml IFN-γ only or stimulated with 12.5 ng/ml IFN-γ and 5μM DAPT for 6 hrs. After stimulation the cells were harvested, RNA isolation was performed and the expression of IFI16 was analysed with RT-PCR. B-D) The macrophages were stimulated on day 8 with a reduced IFN-γ concentration of 5ng/ml or together with DAPT and batimastat 5 μM for 2 or 6 hrs. B) Shows the IFI16 expression C) the M2 macrophage gene Arg1 expression and D) the M1 macrophage gene Ptgs2 expression after stimulation under the described conditions.

Given that macrophages isolated from *cd44*^*-/-*^ mice are in part deficient in their antiviral/antibacterial response, we needed to explore whether differentiated macrophages depend on CD44 cleavage for their function. It is plausible that the macrophages may have required CD44 at early stages of their generation, as the macrophages isolated from *cd44*^*-/-*^ mice are, after all, born from *cd44*^*-/-*^ stem cells. To distinguish between these two possibilities, we tested differentiated *cd44*^*+/+*^ bone marrow derived macrophages for induction of IFI16 upon block of CD44 cleavage by inhibitors of γ-secretase, DAPT. There was no dependence on CD44 cleavage of interferon dependent transcription of IFI16 under optimized M1 differentiation conditions (Fig 8A). To rule out the possibility that the IFN-γ stimulation was too strong to detect potential effects of the inhibitor the IFN-γ concentration was reduced to suboptimal dose (5 ng/ml) and a second inhibitor of matrix metalloproteinases, batimastat, was included (Fig 8B). At two hours after IFN-γ stimulation there was a non-significant tendency of reduction by cleavage inhibition which might suggest that the stimulation even with the low concentration of IFN-γ was so dominant that the contribution by released CD44-ICD was too low (Fig 8B). To test whether DAPT and batimastat would affect the macrophage polarization status, RT-PCR analyses for the genes Arg1 and Ptgs2 were performed. A reduction of Arg1 and Ptgs2 expression in macrophages stimulated with IFN-γ, DAPT and batimastat compared to macrophages stimulated with IFN-γ only was observed, especially at 6 hrs after stimulation (Fig 8C and 8D). This may however not be related to CD44 cleavage (see Discussion).

## Discussion

### The complex role of CD44 in the organism and in the immune system

To define the role/mechanism of the transmembrane glycoprotein CD44 in the organism is made very difficult by its gene structure, transcription and posttranscriptional regulation. Not only is CD44 expressed in numerous cell types, including all cells of the immune system [38] that themselves exert various cellular functions, CD44 occurs in multiple isoforms, protein modifications, and it interacts with different partner proteins (reviewed in [39–41]). Alternative RNA splicing yields a large number of CD44 molecules with different sequences in the extracellular domains. Often in reports on expression and putative CD44 function in cellular systems the isoform expressed has not been examined. Some CD44 functions are mediated by interaction with the major ligand of CD44, hyaluronan. Hyaluronan is synthesized by many cells of the body. It exerts different effects depending on its molecular size. For instance, high molecular weight hyaluronan bound to CD44 prevents tumorigenesis [42], while low molecular weight hyaluronan, often produced by inflammation, induces, independently of CD44, through toll-like receptor 4 the synthesis of defensins [43], activates dendritic cell maturation [44] and the expression of interleukin-8 in endothelial cells [45]. Independently of hyaluronan CD44 interacts with and activates growth factor receptors [23,46].

Deletion of the *cd44* gene in the mouse reveals the complexity and some of the diverse functions in the immune system and its cellular interactors. A total deletion of *cd44* affects of course various cells other than immune cells, which complicates the interpretations. Neutrophil cell migration was enhanced and aggravated in *Escherichia coli* induced pneumonia [12]. In contrast, immunoneutralization of CD44 reduced the neutrophil infiltration in septic lung infiltration upon coecum ligation and puncture [17]. Deletion of *cd44* ameliorated meningoencephalitis induced by *Cryptococcus neoformans* [18]. Pneumonia induced by high doses of *Streptococcus pneumonia*e, however, was not affected by absence of CD44 [12]. It has been suggested that this is due to the expression of hyaluronidase by these bacteria [41]. Macrophages are key players of the innate immune response and several studies have suggested that *cd44* deficiency causes a defective-macrophage phenotype: Inhalation of LPS causes recruitment of macrophages to the lung, but not so in mice with deletion of *cd44* [14]. Isolated *cd44*^*-/-*^ macrophages were less motile and produced less TNF-α. Others examining *Escherichia coli*-induced sepsis found that the isolated *cd44*^*-/-*^ macrophages secreted more cytokines than those from wild type mice [16]. Infection of *cd44*^*-/-*^ bone marrow derived macrophages by *Listeria monocytogenes*, but not by *Salmonella typhimurium*, led, in comparison to WT macrophages, to strongly reduced bacterial intracellular proliferation, paralleled by severely reduced or delayed induction of IL-1, of several other cytokines and of iNOS [19], thus suggesting that CD44 promoted bacterial multiplication. To make sense of these different effects of CD44 deletion would require cell-type specific gene disruptions in the organism and knock-in experiments with different CD44 isoforms/mutants/cleavage products, which have yet not been done.

Our study here addresses the induction of IFI16 transcription in different cell types in culture. The absence of CD44 caused a diminished proinflammatory response and reduced competence of *cd44*^*-/-*^ cells. Enhanced transcription of IFI16 required CD44 cleavage in conjunction with inflammatory inducers. The released CD44-ICD sufficed to rescue transcriptional enhancement in CD44-negative tumor cells and in *cd44*^*-/-*^ MEFs. The hyaluronan binding extracellular domain was not required for the observed induction of IFI16 and IFNβ, nor was any other extracellular or transmembrane CD44 domain needed. In differentiated macrophages we could not obtain significant data (only a tendency) for a requirement of CD44 cleavage in IFI16 induction. Possibly the generation of *cd44*^*-/-*^ macrophages from hematopoietic stem cells depends on CD44 functions other than those we observed in the induction of IFI16, explaining the reduced macrophage functions we observed. The induction by IFN-γ of macrophage differentiation genes was however sensitive to cleavage inhibition. Because macrophage differentiation and polarization depends on Notch signaling [47], we cannot distinguish whether also CD44 cleavage is involved. Inhibition of the Notch-signaling pathway by DAPT could lead to reduced expression of the M1 macrophage specific gene Ptgs2 6 hrs after stimulation with IFN-γ, DAPT and batimastat. Taken together, an attempt to interpret how the observations on CD44 cleavage in isolated and cultured cells translate into an organismic response, requires still major efforts.

### The function of IFI16

IFI16 is an intracellular sensor of DNA in the cytoplasm [22,48], it causes numerous reactions after viral or bacterial entry. Bacterial DNA released into the cytoplasm, e.g. after vacuole destruction by Listeria monocytogenes, causes IFI16-dependent interferon-β expression [35]. IFI16 inhibits the replication of cytomegalovirus [49,50], of HSV1 [51] and of HIV-1 [52]. siRNA dependent downregulation of IFI16 broke the latency of Epstein-Barr virus [53].

### How does the CD44-ICD enhance IFI16 transcription?

To upregulate IFI16 expression, the transcriptional action of the CD44-ICD generated by cleavage suffices. The ICD is transported to the nucleus. The hyaluronan-binding extracellular domain is not required for the observed induction of IFI16 and subsequently of IFN-β. In our microarray study several interferon-inducible genes were upregulated by the CD44 tail. Recently, analysis by Gene Chip microarray yielded 251 genes regulated by the CD44-ICD [54]. EMSA and ChIP data suggest that the CD44-ICD can bind to a DNA element directly, next to the Runx binding site in the MMP9 promoter [54]. An earlier report had suggested that CD44-ICD regulates TPA-dependent genes carrying an AP-1 binding site [1]. Common to interferon-inducible genes is an enhancer sequence called the Friedman-Stark box [55]. However, also IFI16 induction requires the transcription factor AP-1 [28]. Transcriptional co-regulation in B-cells and in-silico analysis to identify transcriptional targets suggest IFI16 regulation by STAT3 and REL [56]. In mammary tumor cell lines downregulation of CD44 reduced and overexpression of CD44-ICD enhanced the expression of the stemness factors Nanog, Sox2 and Oct4 [57]. The stemness factors rely also on the CD44-ICD by direct interaction for nuclear uptake. It is not clear how the transcriptional activation by the CD44-ICD of these genes is accomplished. Because the CD44-ICD carries no recognizable transactivation domain, it is likely that the ICD makes use of a neighboring bona-fide transcription factor, perhaps stimulating complex formation with other regulatory factors of transcriptional initiation.

## Conclusion

Our data indicate a role of CD44 cleavage and release of the CD44-ICD in transcriptional regulation of genes relevant for the antiviral and antibacterial defense. Our *in vitro* results need to be complemented by infection experiments *in vivo*. The data do not exclude that the non-cleaved membrane-bound CD44 molecule plays additional immune cell relevant roles outside of the nucleus, e.g. in cell-cell interactions and cellular migration.
